# A real-world study on the effectiveness of BBIBP-CorV and CoronaVac in Nanjing area

**DOI:** 10.1038/s41598-023-48989-3

**Published:** 2023-12-06

**Authors:** Min Huang, Lu Jia, Sheng Ye, Rongrong Pang, Chengping Ma, Jiajuan Zhang, Shuming Dai, Ke Zhang, Yudong Dai, Qiang Fu, Libo Zhang

**Affiliations:** 1 Department of Blood Screening Laboratory, Nanjing Red Cross Blood Center, Nanjing, Jiangsu China; 2 Department of Quality Management, Nanjing Red Cross Blood Center, Nanjing, Jiangsu China; 3 Apheresis Department, Nanjing Red Cross Blood Center, Nanjing, Jiangsu China; 4 Department of Blood Source Management, Nanjing Red Cross Blood Center, Nanjing, Jiangsu China; 5 Blood Center Management Office, Nanjing Red Cross Blood Center, Nanjing, Jiangsu China

**Keywords:** Infectious diseases, Vaccines, Disease prevention, Public health

## Abstract

Since the advent of COVID-19 vaccine, the long-term monitoring and evaluation of vaccine effectiveness worldwide has never stopped. Real-world research of the mainstream vaccines in China (BBIBP-CorV and CoronaVac) is extremely valuable as a supplement to clinical research data. Venous blood of this study was collected from 111 blood donors and from 6 volunteers, who had received 2 doses of SAR-CoV-2 vaccine. Cross-sectional study and cohort study was adopted. Venous blood of 11 COVID-19 convalescent plasma donors was collected as a positive control. The seroconversion rate of neutralizing antibodies in 111 vaccine recipients was 90.99% (101/111); The level of SAR-CoV-2 antibodies peaked around 28 days after inoculation, then fast descended followed by gentle descended until it was still detectable around 280 days later. The changes in antibody levels were similar to those of the 6 participants and those of convalescent plasma donors after infection. 5 of the 6 participants still maintained a high level of neutralizing antibodies (> 60% of the peak value) around 28 days after receiving 2 doses of vaccine; one participant had an antibody reaction that was almost always negative for 4 weeks. BBIBP-CorV and CoronaVac can produce good immune effects in most vaccinators aged 20 to 59 years in Nanjing area. Nevertheless, significant individual discrepancies of the humoral immunity are still existed.

## Introduction

Since the global pandemic of SARS-CoV-2 (severe acute respiratory syndrome coronavirus 2) in December 2019^1,2^, the centers for disease control and prevention (CDC) and the world health organization (WHO) has proposed various countermeasures against this highly infectious novel virus, among which COVID-19 (coronavirus disease 2019) vaccination is an emergent and significant means to prevent COVID-19 infection and reduce the severity rate^3,4^. Several COVID-19 vaccines have been approved and immunized globally to develop the herd immunity against COVID-19^5^.

In December 2020, several domestic COVID-19 vaccines had been authorized for emergency use in China, two of which were widely used at present: BBIBP-CorV (Sinopharm) and CoronaVac (Sinovac Biotech). Both BBIBPCorV and CoronaVac are inactivated vaccines, the pathogen antigens in which can stimulate the human immune system to produce corresponding antibodies. These two COVID-19 vaccines have been approved for emergency use by WHO. The former is the first COVID-19 vaccine approved for marketing in the world, and the data shows that its protection rate is 79.34%^6^. The vaccines administered by the participants in this study included the above two vaccines.

Generally, it takes more than 10 years to launch new vaccines because it requires a long time to monitor the safety, protective effects and a consistent vaccine administration level. With the successful development and use of COVID-19 vaccines in some countries, the monitoring of COVID-19 vaccines effectiveness and long-term immunity are still desperately demanded not only to prevent its spread but also to restore social and economic activities via generating herd immunization^7^. Monitoring the dynamics of human humoral immune response to COVID-19 vaccination is extremely significant for developing vaccination strategies. The immunological response to COVID-19 vaccination is often evaluated by monitoring the presence of neutralizing antibodies (NAbs) and total antibodies (TAbs)^8,9^.

Although clinical trials can reflect the effectiveness of vaccines, the outcomes are partly dependent on the status of participants. Thus, the data were not very objective. The real-world study can help to establish clinical trial evidence and provide information for adjusting the vaccination strategy^10^.

Therefore, plasma from 111 blood donors of Nanjing Red Cross Blood Center and 6 participants in this study who had completed two-dose BBIBP-CorV and CoronaVac vaccinations were collected for detecting and analyzing the effectiveness of the vaccine via the kinetics of antibody reaction. Convalescent plasma (CP) samples from 11 COVID-19 patients were collected for detecting as a reference.

## Materials and methods

### Ethics approval and consent to participate

This study conducted in accordance with the ethical standards set down by the declaration of Helsinki with its recent modification of 2013 (Fortaleza)^11^ was approved by the Ethics Committee of Nanjing Red Cross Blood Center (NJRCBCEC-2020-01). All participants have filled in the form of blood donor health consultation and signed the informed consent form of blood donors in writing before participating. The informed consent form of blood donors clearly states: "I agree to store the remaining blood samples tested in the laboratory and health-related information into the biological sample bank of healthy people in Nanjing Red Cross Blood Center, which can be used for human medical undertakings in the future." Therefore, the Ethics Committee of Nanjing Red Cross Blood Center allowed this study to be free of informed consent. The data involved in this study were only anonymous screening results, and did not mention any information related to the privacy and identity of blood donors. The blood screening laboratory of this study has passed the review of China National Accreditation Commission Service for Conformity Assessment (CNAS) for medical laboratory in accordance with ISO15189. All staff of the laboratory has signed the confidentiality statement on the privacy and results of blood donors, which required that the privacy and identity information of blood donors be kept confidential.

### Sample sources

To study changes in antibody production, from March 2021 to September 2021, venous blood was collected and detected from 111 unpaid blood donors who had completed the whole process of vaccination (inactivated SARS-CoV-2 vaccine). All blood donors will undergo health consultations before donating, and donors with mild clinical symptoms or diagnosed with COVID-19 infection in the past 6 months are not allowed to donate blood or to be collected venous blood. In order to further explore the antibody reaction kinetics, we also collected venous blood samples from 6 volunteers who received 2 doses of vaccine at 5 time points (before vaccination, 1 week, 2 weeks, 3 weeks and 4 weeks after the second vaccination). Plasma samples from 11 COVID-19 convalescent donors were also collected as the reference, including 1 had received 2 doses of COVID-19 vaccine.

### Cross-sectional study of antibody response of 111 vaccinators

111 unpaid blood donors who had been received two injections of inactivated SARS-CoV-2 vaccine were recruited in the study. Venous blood was collected only once, and the duration from completion of the second vaccination to collection was counted. According to discrepant duration, the above donors were divided into 5 groups: 1–14 days, 15–28 days, 29–60 days, 61–90 days and 91–280 days after the second inoculation. SARS-CoV-2 TAbs, spike-specific IgA, IgG and IgM antibodies at each time point were detected with a chemiluminescent assay (CMIA) kit according to the manufacturer’s instructions (Xiamen Innodx Biotechnology Co., Ltd., Xiamen, Fujian, China). Levels of these antibodies were assessed using qualitative results and S/CO values were obtained. A value ≥ 1.0 was qualitatively defined as positive. The concentration of NAbs were measured in μg/ml as the quantitative results, which Cut-off value is 0.1 µg/ml [COVID-19 neutralizing antibody test kit (magnetic particle chemiluminescence method); Xiamen Innodx Biotechnology Co., Ltd., Xiamen, Fujian, China].

### Cohort study of dynamics of antibody response to vaccination

In order to further explore the kinetics of antibodies production after vaccine administration, the cohort follow-up study was adopted. Venous blood samples were collected at 5 time points (before vaccination, 1 week, 2 weeks, 3 weeks and 4 weeks after the second vaccination) from 6 study participants who received 2 doses of SARS-CoV-2 vaccine. SARS-CoV-2 TAbs, spike-specific IgG and IgM at each time point were detected with the test kit as mentioned above. The concentration of NAbs were measured in μg/ml as the quantitative results with the test kit as mentioned above, and the inhibition ratio of NAbs was calculated according to the test results of another neutralizing antibody reagent(cPass™ SARS-CoV-2 neutralization antibody detection kit; GenScript USA,Inc.,USA).According to the reagent instructions, the inhibition rate formula was as follows:

$${\text{Percent Signal Inhibition }} = \, [1 - \left( {{\text{OD value of Sample/OD value of}}\;{\text{Negative Control}}} \right)] \times 100\%$$

### Antibody response to infection with SARS-CoV-2 of the CP donors

Plasma samples from 11 COVID-19 convalescent plasma donors were collected, including 1 had received 2 doses of SARS-CoV-2 vaccine. The duration of the 11 patient's disease course was counted and used as the reference for the duration from completion of 2 doses of vaccine to collection. SARS-CoV-2 TAbs, spike-specific IgA, IgG and IgM antibodies at each time point were detected with a CMIA kit as mentioned above. The concentration of NAbs were measured in μg/ml as the quantitative results (COVID-19 neutralizing antibody test kit as mentioned above).

### Statistical analysis

Essential information and composition ratio of various characteristics of the 111 vaccinators was collected and calculated. The testing results of blood samples were automatically derived by laboratory software. All the charts and diagrams were drawn using R software (R, version 4.2.0).

## Results

### Characteristics of 111 vaccinators

111 blood donors who had been received two injections of inactivated SARS-CoV-2 vaccine were recruited in the study. Demographic condition data of the vaccine recipients were summarized in Table 1, containing various blood types, education and career. 80(72.07%) males and 31(27.93%) females were included, with a median age of 36 years. The constituent ratio of university education (36.04%) and the career of office worker (35.14%) was highest. The seroconversion rate of neutralizing antibodies in 111 vaccine recipients was 90.99% (101/111) (Supplementary Table S1A).

**Table 1:** Characteristics of 111 vaccinators.

Characteristics		Vaccine recipients (n = 111) Number (constituent ratio)
Gender	Male	80 (72.07%)
	Female	31 (27.93%)
Age	20–29	37 (33.33%)
	30–39	27 (24.32%)
	40–49	26 (23.42%)
	50–59	21 (18.93%)
Blood type	A	38 (34.23%)
	B	32 (28.83%)
	O	31 (27.93%)
	AB	10 (9.01%)
Education	Junior school	6 (5.40%)
	Vocational School	13 (11.71%)
	Senior school	13 (11.71%)
	College	24 (21.62%)
	University	40 (36.04%)
	Graduate school	12 (10.81%)
	Unknown	3 (2.70%)
Career	Worker	9 (8.11%)
	Civil servant	2 (1.80%)
	Teacher	3 (2.70%)
	Farmer	2 (1.80%)
	Student	14 (12.61%)
	Medical staff	11 (9.91%)
	Office worker	39 (35.14%)
	Unknown	31 (27.93%)

### Antibody levels and trends of 111 vaccinators

**Figure 1:**
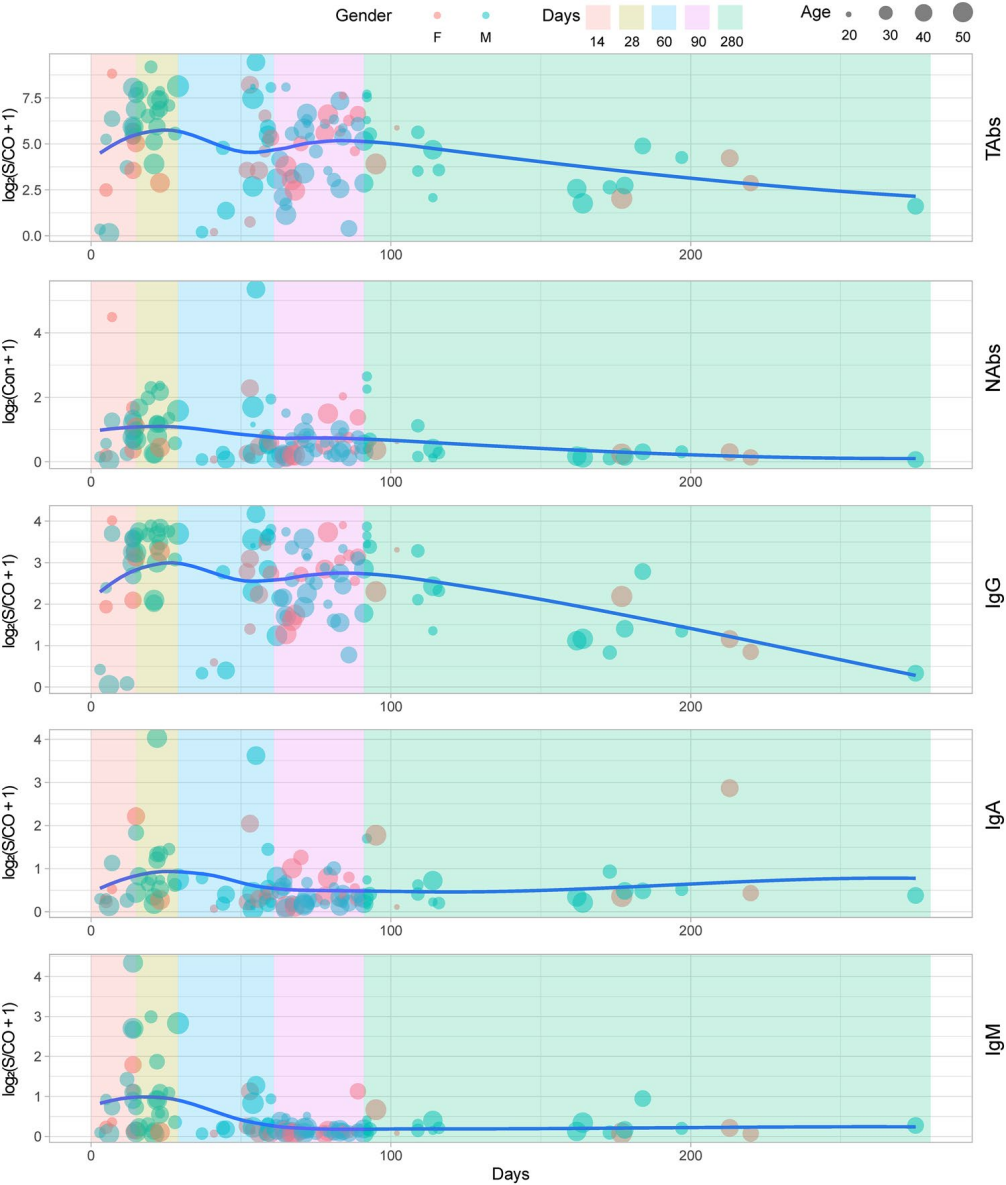
Antibody levels and trends of 111 vaccinators. log_2_(S/CO + 1), logarithmic conversion result of S/CO; log_2_(Con + 1), logarithmic conversion result of concentration; F, female; M, male; Day, days from the second dose of vaccine to collection; 14, 1–14 d; 28, 15–28 d; 60, 29–60 d; 90, 61–90 d; 280, 91–280 d;20, 20–29 years old; 30, 30–39 years old; 40, 40–49 years old; 50, 50–59 years old. TAbs, total antibodies; NAbs, neutralizing antibodies.

111 blood donors were divided into 5 groups according to the duration from completion of the second vaccination to collection, which ranged from 3 to 275 days (Supplementary Table S1A) as presented with 5 different colored blocks in Fig. 1. A bubble represented a vaccine recipient. The ordinate of Fig. 1 was obtained by logarithmic conversion of the detection results (S/CO or concentration) of antibody level. The longitudinal position of the bubbles corresponded to different ordinate values. As shown from the curve which represented the trend of bubbles clusters over time in Fig. 1, a rise within 28 days could be observed. Levels of SARS-CoV-2 antibodies were gradually decreases thereafter and could still be detected until about 280 days. Although the results of IgA and IgM were negative in most vaccinators, the results of S/CO indicated no significant change in their levels, as shown in Supplementary Table S1A and in Fig. 1. Ages of these vaccination recipients were divided into 4 groups: 20–29, 30–39, 40–49, 50–59. They were represented by four discrepant sizes of bubbles, from small to large. Genders of these vaccination recipients were represented by different bubble colors, as shown in Fig. 1. The distribution of gender and age in discrepant groups among five antibodies was similar.

### Dynamics of antibody response to vaccination of 6 participants

**Figure 2:**
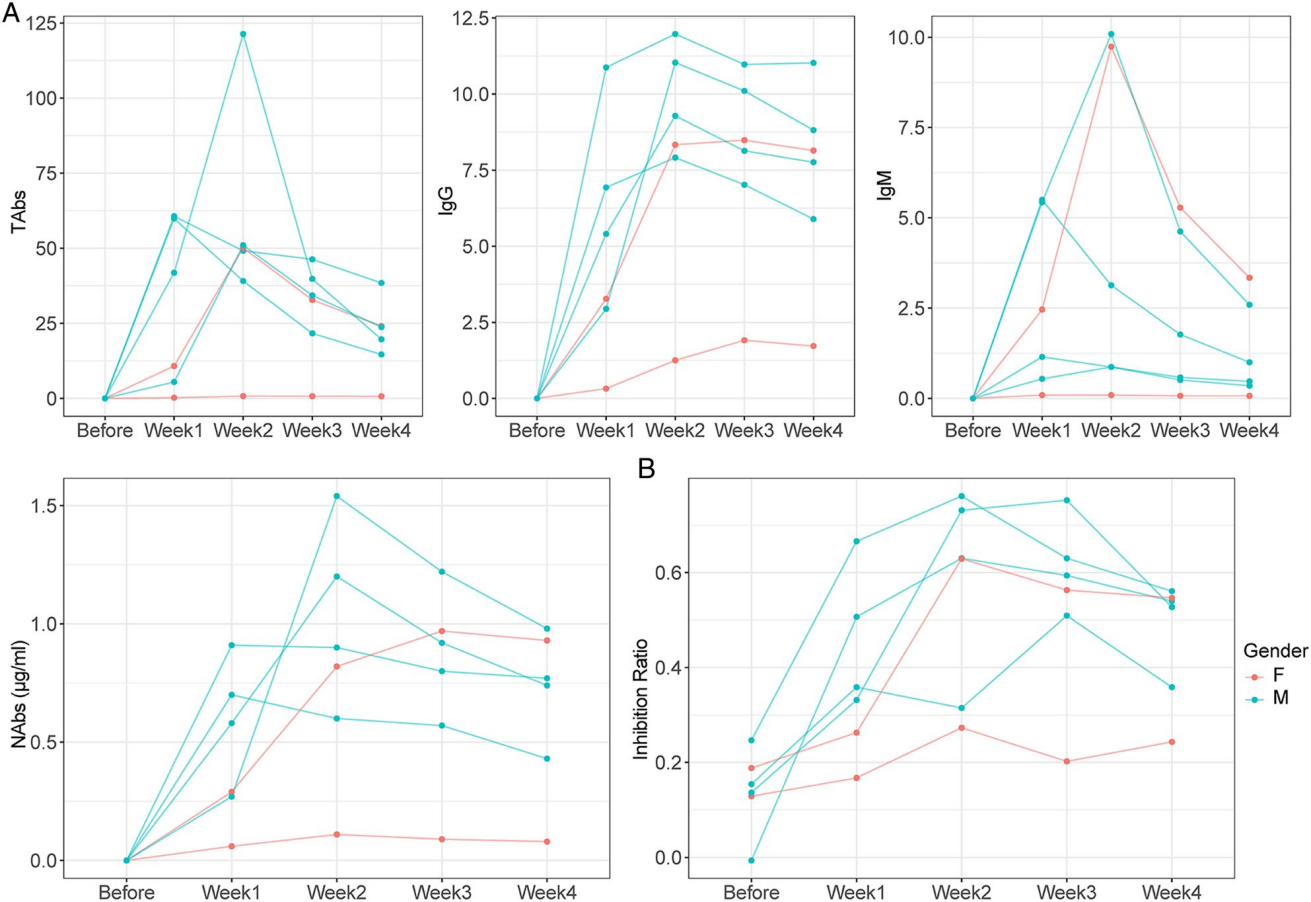
Dynamics of antibody response to vaccination of 6 participants. (**A**) The antibody [TAbs (S/CO), NAbs (concentration), IgG (S/CO), IgM (S/CO) and IgA (S/CO)] levels induced by 2 doses of SARS-CoV-2 vaccines at several time points as before vaccination, 1 week, 2 weeks, 3 weeks, 4 weeks after vaccination; (**B**) Changes of inhibition ratio of the NAbs at various time points. Inhibition Ratio, inhibition ratio of the NAbs; F, female; M, male. TAbs, total antibodies; NAbs, neutralizing antibodies.

The kinetics change of the participants was variable and most of which presented a bell-shaped curve. The SARS-CoV-2 TAbs of 2 out of 6 participants (2/6) peaked in the first week and 3 (3/6) in the second week (Fig. 2). IgM showed a similar shape to TAbs. In contrast, IgG appeared later, 4 out of 6 participants (4/6, male) peaked at week 2 and 2 (2/6,female) peaked at week 3. NAbs of 2 out of 6 participants (2/6, male) peaked in the first week and 2 (2/6, male) in the second week and 1 (1/6, male) in the third week. According to the reagent specification, the inhibition ratio is proportional to the antibody concentration. As shown in the NAbs inhibition ratio curve (Fig. 2B), NAbs inhibition ratio of 3 out of 6 participants (3/6) peaked in the second week and 2(2/6) in the third week. Notably, most antibodies from 1 recipient (1/6, female) was found to remain negative at all follow-up time points (Supplementary Table S1B), and the dynamic curve appeared as a straight Line.

### Antibody response to infection with SARS-CoV-2 of 11 CP donors

**Figure 3:**
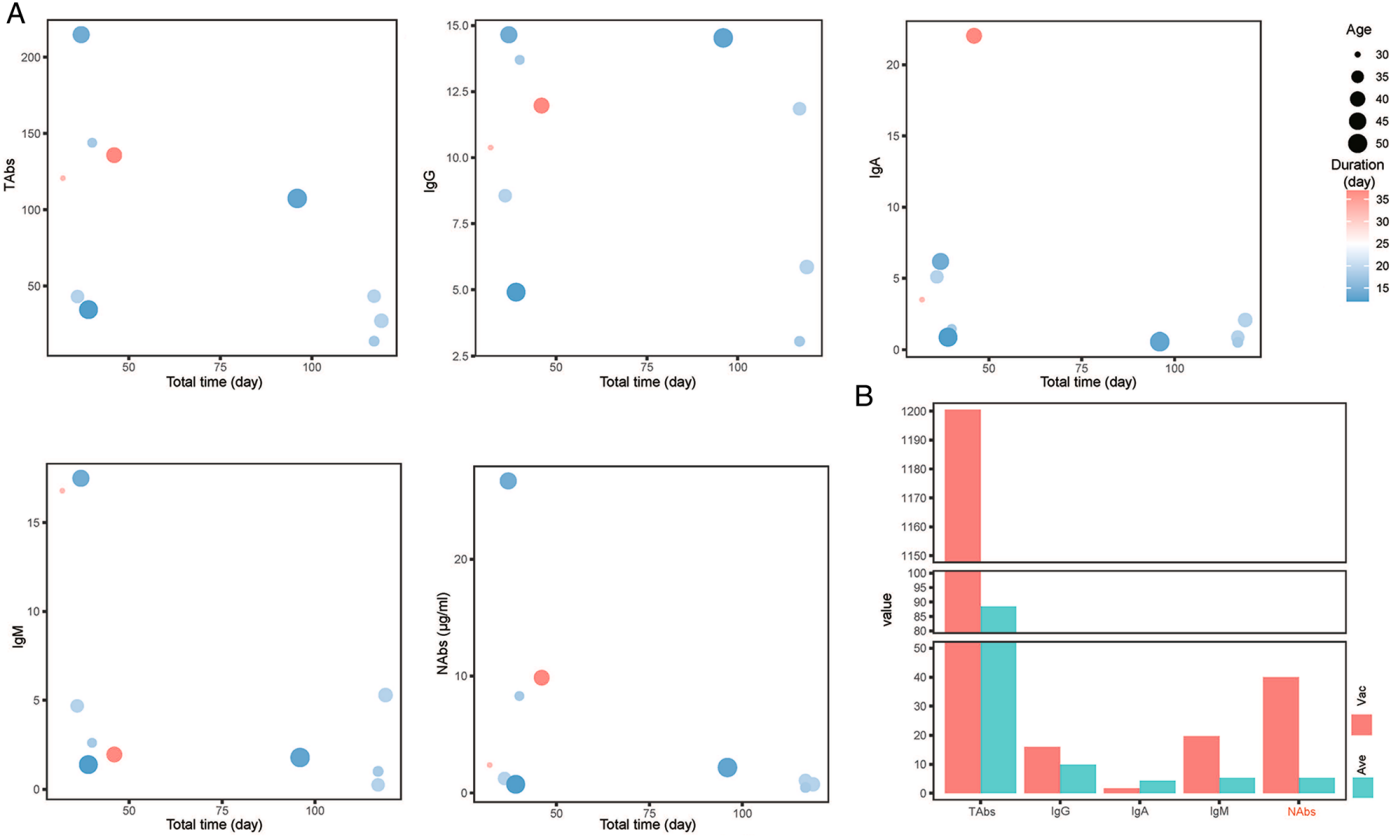
Antibody response to infection with SARS-CoV-2 of 11 CP donors. (**A**) Antibody production in 10 non-vaccinated CP donors; (**B**) Antibody levels of 1 case of CP donor vaccinated before infected with SARS-CoV-2 were compared with the average values of other non-vaccinated CP donors. Total time, the time from infection to collection; Duration, the course of disease; Vac, antibody levels of the CP donor with two doses of vaccination completed; Ave, average antibody levels of the remaining 10 non-vaccinated CP donors. Value, semi quantitative test results of antibodies (TAbs, IgG, IgA, IgM) expressed as S/CO, or quantitative test results of NAbs expressed as concentration (μg/ml). TAbs, total antibodies; NAbs, neutralizing antibodies.

Blood samples from 11 CP donors were also collected and did the same tests. Among the 10 non-vaccinated CP donors, total time from infection to collection spanning the course of disease(duration) and the course from recovery to collection (interval) ranged from 32 to 119 days. Except for IgG, the antibody levels presented a gradually decreased trend with the total time. As shown in Fig. 3A, every bubble represented a non-vaccinated CP donor and its sizes represented different age groups (30–34, 35–39, 40–44, 45–49, 50–54), while its colors represented various lengths of disease course(duration) ranged from 12 to 37 days (Supplementary Table S1C). 1 CP donor had been finished the whole vaccination before. Individual infected after vaccination showed much higher antibody levels and NAbs concentration (Fig. 3B).

## Discussion

The kinetic changes of antibodies in this study were mainly observed in cohort study data (Fig. 2). Cross-sectional study data could be considered as a reference (Fig. 1), and CP donors’ data might be regarded as a positive control (Fig. 3). NAbs and TAbs are good indicators for evaluating COVID-19 vaccine. NAbs levels are generally used as the primary surrogate endpoint of the immunological protection of viral vaccines^12^.

The curve of the bubble diagram (Fig. 1) showed that the various antibodies of 111 blood donors after receiving 2 doses of vaccine showed relatively higher levels around 28 days, and then gradually decreased. The NAbs remained at a medium to high level (> 70% of the peak value) for about 90 days, and had steadily declined since then. It could still be detected after about 280 days. The seroconversion rate of NAbs in this population was 90.99% (101/111), indicating that the current mainstream vaccines BBIBP-CorV and CoronaVac in China were protective. It has been reported that the seroconversion rate of NAbs against BBIBP-CorV vaccine was 90.7%^9^, and 77.9% (18–59 years old) of NAbs against CoronaVac vaccine were seroconverted by 28 days after the second vaccine dose^7^. Due to the limitations of the cross-sectional study, the seroconversion rate of NAbs in 111 blood donors in this study was likely to be underestimated, and the antibody dynamics of each individual was not complete, but this result was close to the results of relevant reports and could roughly outline the distribution of COVID-19 antibody levels in 111 blood donors in discrepant periods.

The kinetic curve of NAbs (Fig. 2) showed that 1 of the 6 participants had a very low NAbs level, displaying a weak positive reaction only at 14 days, while the remaining time points were negative. The curve was almost horizontal and straight, and the NAbs inhibition rate of this participant was negative (< 30%) for 4 weeks. This suggests that in the real-world study, the seroconversion rate of NAbs is difficult to reach 100%. Due to individual differences, some individuals have low immune response levels, and vaccination still cannot effectively prevent infection. Even so, the probability of severe infection in these people is much lower^13^.

The NAbs levels of the remaining 5 participants reached their peak among 7–21 days, and then decreased slowly; In the 4th week, the NAbs levels still remained above 60% of the peak value, which meant that the NAbs levels remained high at 28 days after receiving two doses of vaccine (Fig. 2A). This is similar to the dynamics of NAbs in patients with COVID-19^14^. As can be seen from Fig. 1, serum neutralizing activity after infection or vaccination showed a biphasic decline. Following an initial drop that is also driven by the reduction in IgM and IgA antibodies with shorter half-life^15^, the titer decay of NAbs since then was slower and the serum neutralizing activity of most individuals could still be detected for more than 1 year, although the level is relatively low^15,16^.

The NAbs inhibition rate result (Fig. 2B) showed that the NAbs inhibition rate reached a peak within 2–3 weeks, and then gradually decreased until the 4th week, maintaining a medium to high level. It was found that for an individual regardless of the whole, the NAbs inhibition rate and the kinetic changes in NAbs concentration (Fig. 2A) were not consistent. This might be related to diverse experimental methods, indicating the presence of considerable differences in the measured NAbs levels of the same sample among discrepant laboratories and methods^17,18^. Meanwhile, NAbs directed at regions outside of the RBD (receptor binding domain) will not be detected by reagents using the RBD as the target antigen. It cannot, therefore, be assumed that the activity in one type of assay, such as neutralization, strictly parallels another. Even between binding assays, antibody titers against two viral proteins (e.g., nucleoprotein and S protein) might not necessarily correlate^19^.

The IgM of 6 participants appeared at the earliest stage, reaching a peak within 7–14 days, and then decreased rapidly. After 4 weeks, 4 out of 5 participants decreased by more than 50% (Fig. 2A). IgG appeared relatively late, reaching its peak among 14–21 days, and then gradually decreased. Until 4 weeks later, IgG antibody levels remained above 70% of their peak levels. Both IgG and NAbs could maintain medium to high levels for a long time after vaccination, but the changes in their kinetic curves were inconsistent. In 10 CP donors, inconsistencies between the two could also be seen, with a significant difference in the distribution of NAbs and IgG levels around the 100th day of the total time after infection (Fig. 3A). This may be due to the inconsistent classification methods of the two type antibodies and the existence of a crossover effect between their antigen-binding sites. Unlike the characteristic that NAbs mainly target RBD, SARS-CoV-2 spike-targeting IgG reactivity in convalescent and vaccinated individuals is largely directed to epitopes outside of the RBD and many S-reactive antibodies do not neutralize the virus^19,20^.

Since the blood sample of each CP donor was collected only once, the results in Fig. 3 could also be considered as a cross-sectional study. Figure 3A showed that the total time from infection to collection for 10 CP donors was between 32 and 119 days, which was inversely proportional to the NAbs levels and showed an overall downward trend. 6 CP donors had a total time between 30 and 50 days after infection, with high antibody levels. 4 CP donors had a total time above 90 days after infection, with weak antibody levels, but they were still positive. The total time from vaccination to collection (Figs. 1, 2) could be assumed as a positive control. The antibody dynamics changes after vaccination in this study (Fig. 1) were similar to this, and also similar to the results reported in relevant reports^21^. It is suggested that the widely used vaccines had good immune effects.

The length of the course of disease to some extent reflects the severity of clinical symptoms. Some studies have shown that high titer levels of antibodies in patients are independently associated with a worse clinical classification^22^. Similar to previous reports in Fig. 3A, except for one specific data, the antibody levels of the remaining individuals had a positive correlation with the duration of the disease.

Due to the limitations of the study subjects, this study only analyzed the antibody levels of individuals aged 18–60 years. Neither Fig. 1 nor Fig. 3 showed the correlation between age and antibody levels. But studies have shown that, the NAbs titers in young vaccine recipients had a significantly higher peak than those in elderly recipients. In addition, the Phase I trial showed that the serum titer of subjects > 60 years old after 28 days of the second dose was less than that of subjects aged 18–59, indicating that the elderly may need higher doses or adjuvants with stronger immunogenicity ^9,10^.

Compared with the male participants, the female antibody levels especially NAbs and IgG, rose later, the levels were lower, and even there was no antibody reaction, suggesting that the antibody reaction of women to COVID-19 might be weaker than that of men (Fig. 2). Nevertheless, this discrepancy was not significant in the population (Fig. 1).

It is worth noting that the levels of TAbs, NAbs, and other antibodies in 1 of the 11 CP donors was extremely higher than those of the other 10 non-vaccinated CP donors. That means, more antibodies could be produced when infected with SARS-CoV-2 after vaccination. Some studies suggest that the high antibody levels during the convalescent period when the vaccine is superimposed is related to an increase in the titer and the breadth of targeting virus variants caused by increased doses^20,23^.

Finally, there are still several limitations in this study: Firstly, there are partial cross-sectional research data in this study, with fewer cohort studies, and a lack of individual dynamic data with large sample size; Secondly, the vast majority of the subjects in this study are blood donors, aged between 20 and 59 years old, and there was no evaluation on the children and the elderly; Thirdly, the prevention effect of the vaccines involved in this study on variants such as Omicron were not evaluated^10,24^; Finally, this study has only evaluated the effectiveness of vaccines in humoral immunity, without involving cellular immunity. Currently, we are conducting further evaluations of memory B cells and cellular immunity.

## Conclusions

The results of this study have shown that the two mainstream vaccines BBIBP-CorV (Sinopharm) and CoronaVac (Sinovac Biotech) in China can trigger an immune response and produce good immune effects in most vaccinators aged 20 to 59 years. When infected with SARS-CoV-2, antibodies can be produced more in the vaccinators than the non-vaccinators. We also found that, dynamics of the NAbs response in individuals who have been vaccinated with SARS-CoV-2 vaccine or recovered from COVID-19 vary greatly, and prediction of immune longevity can only be accurately determined at the individual level^15^, and evaluation of vaccine effectiveness must be based on a population level.

### Supplementary Information

Supplementary Information.

## Data Availability

The data presented in this study are available in this published article and its supplementary files [S1].

## Abbreviations

COVID-19: Coronavirus disease 2019
CDC: The Centers for Disease Control and Prevention
WHO: World Health Organization
SARS-CoV-2: Severe acute respiratory syndrome coronavirus 2
NAbs: Neutralizing antibodies
Tabs: Total antibodies
IgG: SARS-CoV-2 spike-specific IgG
IgM: SARS-CoV-2 spike-specific IgM
CP: Convalescent plasma
RBD: Receptor binding domain
CMIA: Chemiluminescence microparticle immuno assay
